# Autophagy is associated with chemoresistance in neuroblastoma

**DOI:** 10.1186/s12885-016-2906-9

**Published:** 2016-11-15

**Authors:** Assila Belounis, Carine Nyalendo, Roxane Le Gall, Tina V. Imbriglio, Mohamed Mahma, Pierre Teira, Mona Beaunoyer, Sonia Cournoyer, Elie Haddad, Gilles Vassal, Hervé Sartelet

**Affiliations:** 1 Research centre of the Sainte Justine university hospital, Montreal, QC Canada; 2 Department of pathology and cellular biology, Université de Montréal, Montreal, QC Canada; 3 Department of biochemistry, CHU Sainte Justine, Montreal, QC Canada; 4 Division of paediatric oncology, CHU Sainte Justine, Montreal, QC Canada; 5 Department of surgery, CHU Sainte Justine, 3175 Montreal, QC Canada; 6 Department of paediatric oncology, Institut Gustave Roussy, Villejuif, France; 7 Department of pathology and cytogenetic, CHU Sainte Justine, Montreal, QC Canada

**Keywords:** Neuroblastoma, Autophagy, Chemoresistance, Hydroxychloroquine

## Abstract

**Background:**

Neuroblastoma (NB) is a frequent pediatric tumor characterized by a poor prognosis where a majority of tumors progress despite intensive multimodality treatments. Autophagy, a self-degradative process in cells, could be induced by chemotherapy and be associated with chemoresistance. The aim of this study was to determine whether: 1) autophagy is present in NB, 2) chemotherapy modified its levels, and 3) its inhibition decreased chemoresistance.

**Methods:**

Immunohistochemical stainings were performed on samples from 184 NB patients in order to verify the expression of LC3B, a specific marker for autophagy, and Beclin 1, a positive regulator of autophagy. In addition, we performed an in vitro study with six NB cell lines and six drugs (vincristine, doxorubicin, cisplatin temozolomide, LY294002 and syrolimus). Inhibition of autophagy was performed using ATG5 knockdown cells or hydroxychloroquine (HCQ). Cell survival was measured using the MTT cell proliferation assay. Autophagy was detected by monodansylcadaverine, confocal microscopy and Western blot. In vivo study with tumor xenografts in NSG mice was performed.

**Results:**

Our results have indicated that autophagy was present at low levels in NB and was not a prognostic factor, while Beclin 1 was highly expressed in children with poor NB prognosis. However, autophagy levels increased after chemotherapy in vitro and in vivo. Tumor progression was significantly decreased in mice treated with a combination of HCQ and vincristine.

**Conclusions:**

Taken together, autophagy is present in NB, induced by chemotherapy and associated with chemoresistance, which is significantly reduced by its inhibition. Therefore, targeting autophagy represents a very attractive approach to develop new therapeutic strategies in NB.

**Electronic supplementary material:**

The online version of this article (doi:10.1186/s12885-016-2906-9) contains supplementary material, which is available to authorized users.

## Background

Neuroblastoma (NB) is the most common and deadly extracranial solid tumor in children [1, 2]. Survival of children older than one year of age with advanced NB is poor (only 34%), despite aggressive treatments [3, 4]. High-dose chemotherapy with autologous hematopoietic stem cell transplantation significantly improves the prognosis of metastatic NB [5, 6], but this treatment carries with it a high risk of adverse effects [4]. Poor global survival and resistance to high-dose chemotherapy indicate that NB is specifically associated with chemoresistance [7].

Autophagy is a ubiquitous self-degradation process that involves the degradation and recycling of cellular cytoplasmic constituents through the lysosomal pathway. Damaged or misfolded proteins or organelles are first sequestered in double-membrane vesicles, known as autophagosomes, before fusing with lysosomes, where their contents are degraded by lysosomal proteases [8–10]. Autophagy is a complex and multistep process involving the autophagy-related proteins (ATG) [8]. ATG5 is a protein involved in the early stages of autophagosome formation and plays an essential role in the maturation of autophagosomes [11], with assistance from LC3 [8]. Low levels of autophagic activity are commonly observed under normal conditions, presumably preserving normal cellular homeostasis [12, 13]. Prolonged autophagy may result in type 2 (autophagic) programmed cell death [12, 14]. The activation of autophagy is measured by the ratio between LC3II on the autophagosome membrane and LC3I in the cytoplasm which can be detected by Western-blot [15] or by immunohistochemistry [16]. Beclin 1 is also a marker and a positive regulator of autophagy.

The regulation of autophagy by the PI3K/AKT pathway is very complex. Recently, an AKT inhibitor was reported to induce autophagy with a radiosensitizing effect [17]. Autophagy is regulated by both class I and III PI3K pathways [18, 19]. mTOR serves as a metabolic sensor that coordinates cross-talk between nutrient availability and autophagy [19]. On the other hand, class III PI3K in conjunction with Beclin 1 positively regulates autophagy [18, 19].

In cancer, autophagy plays a dual role by either activating cell death and inhibiting tumor progression or promoting cell survival [20]. In the early stages of carcinogenesis, autophagy acts as a primary tumor suppressor and inhibits tumor progression [21]. However, autophagy can also confer tumor cells the ability to resist to ionizing radiation [22] as well as to chemotherapy [23].

The observation of increased cell survival associated with higher autophagy activity following therapy has led to the development of strategies combining autophagy inhibitors to current anticancer treatments. In this context, chloroquine (CQ) or its derivate hydroxychloroquine (HCQ), sensitizes tumor cells to anticancer therapies. Indeed, CQ and HCQ block the processing and maturation of autophagy vacuoles (autophagolysosomes) by inhibiting lysosomal activity [23]. Some data suggest that autophagy inhibition and autophagosome accumulation both contribute to the accelerated cell death induced by HCQ [23].

The aim of the present study is to demonstrate the presence of autophagy in NB, its activation by chemotherapy and its correlation with chemoresistance.

## Methods

### Study design and patients

Study cases were selected upon the following inclusion criteria; 1) a diagnosis of NB had been made between July 1988 and March 2008, 2) human subject research (tissue samples) was approved by the Research Ethics Board of the Sainte-Justine University Health Center. Written consent has been obtained from patient guardians, and 3) adequate specimen material has been collected for study purposes. 184 patients with NB were included in our study. The patients were treated and followed up at Sainte-Justine University Health Center (Montreal, Canada). Thirty-one out of 184 total NB cases were identified from routine provincial (Quebec, Canada) mass screening efforts. Tumors were classified according to the International Neuroblastoma Staging System (INSS) [24]. Treatment was assigned according to the risk group on the basis of the patient’s age at time of diagnosis, the INSS stage, the histoprognosis, the ploidy and *MYCN* amplification status (v-myc avian myelocytomatosis viral oncogene neuroblastoma derived homolog). With formalin-fixed and paraffin-embedded samples, a tissue microarray (TMA) was constructed using four representative NB tumor tissue cylinders with a 0.6 mm diameter. TMA blocks contained not only 184 primary tumors but also 47 paired metastases (42 lymph nodes and 5 hepatic metastases). Among the 184 tumors, 19 tumors were tested by Western blot, proteins coming from the lysate of frozen samples.

### Immunohistochemistry

Immunohistochemistry was performed on the sections of the TMA blocks or of tumors developed in the mouse model. The Ultraview Universal DAB detection kit (Ventana, Ventana medical system, Tuscon, AR) was used. Antibodies against phospho-AKT (1/100, S473-r, Santa Cruz biotechnology, CA), phospho-mTOR (1/100, 49 F9, Cell Signaling, CA), LC3B (1/1000, ab51520 abcam, Cambridge UK) or Beclin 1 (1/250, ab55878 abcam) were applied for 30 min. DAB was used as a chromogen and hematoxylin as a counterstain. Normal mouse or rabbit IgG at the same concentration as the primary antibody were used as negative control and synaptophysin (1/100, Polyclonal, SP11, Thermofisher Scientific) as positive control (Additional file 1: Figure S1). Two investigators blinded for clinical data independently evaluated immunostaining in samples containing more than 100 NB cells. Immunostaining scores were established by a semi-quantitative optical analysis assessing the percentage of positive cells in each sample: 0 = all cells negative, 1 + = 1 to 25%, 2 + = 26 to 50%, 3 + = 51 to 75% and 4+ more than 75% of positive tumoral cells.

### TUNEL

On the sections of TMA, a terminal deoxynucleotidyl transferase-mediated dUTP nick end-labeling (TUNEL) assay (In situ cell death detection kit, POD (Roche)) was used to identify double-stranded DNA fragmentation, characteristic of DNA degradation due to apoptosis. Briefly, tissue slides were deparaffinized. The slides were then treated with 0.1% of Triton X-100 (Sigma, X-100). The slides were then incubated with terminal deoxynucleotidyl transferase followed by peroxidase-conjugated anti-digoxigenin antibody. Finally, the slides were stained with DAB. Methyl green was performed as the counter-stain. Slides were scanned using a customized, computer-controlled microscope (Axio Imager M1; Zeiss, Oberkochen, Germany). The percentage of positive neuroblasts for TUNEL was also calculated by dividing the number of stained nuclei by the total numbers of neuroblasts and multiplying by 100.

### Cell lines

Six NB cell lines were studied: SK-N-DZ (ATCC, Manassas, VA), SK-N-SH (ATCC), SK-N-FI (ATCC), IGR-N91 and IGR-NB8 cells from Gustave Roussy Institute (Villejuif, France), and NB-10 (St. Jude Children’s Hospital, Memphis, TN). *MYCN* amplification is present in NB-10, SK-N-DZ and IGR-N91 cells. The cells were cultured in Dulbecco’s modified Eagle medium (DMEM), 10% fetal bovine serum (FBS) and 1% penicillin-streptomycin at 37 °C in a 5% CO_2_ atmosphere.

### Knockdown of ATG5 expression by lentivirus-delivered shRNA

TRC Lentiviral Human ATG5 and eGFP shRNA vectors (ATG5: accession #NM_004849, eGFP: accession # RHS4459) were purchased from Open Biosystems, Rockford, IL. Lentiviral vectors were produced using HEK 293 T cells by PEG (polythylenimine linear, Polysciences inc) transfection of ATG5 or eGFP shRNA plasmid together with the third-generation packaging plasmids pMDL, pRev and pV-SVG (Open Biosystems). To generate human ATG5-knockdown cells, IGR-N91 cells were transduced with lentivirus expressing shATG5 or sheGFP for control. Transduced cells were cultured in fresh medium for 2 days before selection for stable expression of the shRNA by growing in culture media containing puromycin (5 μg/mL) for at least 2 weeks.

### GFP-LC3 transfection and confocal microscopy

The cell line IGR-N91 was transfected with GFP-LC3 (Millipore’s LentiBrite TM GFP-LC3 lentiviral Biosensor) for monitoring autophagosome formation. IGR-N91 cells were seeded at 4 × 10^5^/well into eight-well chamber slides (Thermo Scientific, Rochester, NY) to achieve 70% confluence. After 24 h, the cells were transfected with lentivirus containing a version of GFP-LC3 at 37 °C, 5% CO_2_ for 24 h. At 48 h post-transfection, the medium was changed to DMEM, 1% fetal bovine serum (FBS) and 1% penicillin-streptomycin, the cells were washed three times with PBS and visualized with the Ultraview Vox Confocal Imaging System (Perkin Elmer). The autophagosome volume in transfected cells was evaluated with Imaris 7.7.2 software (Oxford Instruments Company).

### Cell proliferation assay

Cell viability was determined by the MTT test. Cells were plated in a 96-well culture plate at a density of 5x10^3^/well over night. The cells were treated with various conditions for 24 h then proliferation was measured by the (3-[4,5dimethyl-2-thiazolyl]-2,5-diphenyl-2H-tetrazolium bromide) MTT cell proliferation assay (Cell Titer 96 Non-Radioactive Cell proliferation Assay, Promega) according to manufacturer’s instructions. Absorbance was measured at 570 nm using the Spectra Max 190 microplate spectrophotometer (Molecular Devices, Sunnyvale, CA). Assays were performed in triplicate. Relative cell viability (percent of control) was calculated using the equation: (mean OD of treated cells/mean OD of control cells) × 100. For each time point, the treated cells were compared with control cells that had been treated with vehicle only.

### Monodansylcadaverine (MDC) test

To correlate cell survival with the presence of autophagic vacuoles, cells were incubated with the autofluorescent agent monodansylcadaverine (MDC) at 0.05 mM in PBS (Sigma) for 10 min at 37 °C. MDC has been reported to specifically label autophagic vacuoles [25]. MDC was then replaced with 200 μl of PBS and finally replaced by 100 μl of Tris-Triton (Tris 10 mM pH 8.0, 0.1% Triton X-100). Fluorescence was measured with EnVision 2104 multilabel reader (Perkin Elmer) with an emission filter of 525 nm at 380 nm.

### Cell treatments

The MTT and MDC tests were performed on cell lines with or without transfection of ATG5 shRNA vector and treated with different concentrations of drugs for 24 h at 37 °C: temozolomide (Schering Plough inc, 0.1–1000 μM), vincristine (Mayne Pharma USA inc, Paramus NJ, 1–10000 nM), doxorubicin (Pfizer Inc Kirkland, Canada, 0.005–50 μM) and cisplatin (Mayne Pharma USA inc, Paramus NJ; 0.015–150 μg/ml), LY294002, specific inhibitor of AKT (Calbiochem Darmstadt, Germany, 0.05–500 μM) and rapamycin, specific inhibitor of mTOR (Pfizer Inc Kirkland, Canada 0.1–1000 nM). Finally, cells were treated with varying concentrations of cisplatin (0.15–75 μg/mL) and doxorubicin (0.005–5 μg/mL) in the presence and absence of HCQ (30 μM) for 48 h. The compound concentrations resulting in 50% inhibition of cell viability (IC_50_) were determined using GraphPad Prism 6 software. The cell line IGR-N91 transfected with GFP-LC3 were treated with vincristine (1 μM), with HCQ (30 μM) or with the association of the two drugs and then analyzed with confocal microscopy for 7 h.

### Western Blot

Protein extracts were prepared from frozen tumor tissues of 19 patients and cultured cell lines. Cell lysis buffer with protease inhibitor cocktail (10 mM Tris–HCl pH 7.4, 150 nM NaCl, 1 mM EDTA, 1 mM EGTA, 1% Triton X-100, 0.5% NP-40, 1 mM sodium orthovanadate, 1 mM sodium fluoride) was added to each sample. Equal amounts of protein (20 μg) from cell lysates were solubilized in Laemmli sample buffer, boiled for 5 min, separated by SDS-PAGE, transferred onto polyvinylidene difluoride (PVDF) membranes, blocked 1 h at room temperature with TBS buffer (20 mM Tris–HCl, pH 7.4, 150 mM NaCl) containing 3% bovine serum albumin, and incubated with primary antibody overnight at 4 °C. Immunoreactive bands were revealed following 1 h incubation with horseradish peroxidase-conjugated anti-rabbit or anti-mouse antibodies, and the signals were visualized with an enhanced chemiluminescence (ECL) detection system (PerkinElmer, Waltham, MA). The primary antibodies used for this study were: β-actin (13E5; diluted 1:5000, Cell Signaling, Danvers, MA), mouse polyclonal anti-human PARP-1 (Ab-2; 1:600; Calbiochem, Billerica, MA), rabbit polyclonal anti-human Beclin 1 (1/1000, ab55878 abcam, Cambridge UK), rabbit polyclonal anti-human SQSTM1/p62, LC3B and ATG5, rabbit monoclonal anti-human Cleaved Caspase-3 (Asp175), mTOR (7C10), phospho-mTOR (Ser2448, D9C2), Akt and phospho-Akt (Ser473), (all diluted 1:1000, Cell Signaling, Danvers, MA). A densitometry analysis with the Kodak ID 3.6 software was used to calculate the relative expression of Cleaved Caspase-3 and the ratio between LC3B-II and LC3B-I.

### Animal experiments

NOD/LtSz-scid/IL-2Rgamma null mice (NSG) were purchased from Jackson Laboratory (Bar Harbor, ME, USA). Animal experiments were approved by the CEEA26 Ethics Committee (approval number: 2013–099) and carried-out under the conditions established by the European Community (Directive 2010/63/UE). All mice were housed in ventilated cages under standard conditions of controlled temperature and humidity, and exposed to a 12-hourly light/dark cycle. They were provided with standard diet and water ad libitum. First, NSG were established in 6 to 8 week-old mice by subcutaneous injection of 5 × 10^6^ IGR-N91 in the left flank. Mice were monitored twice a week for tumor growth. Clinical monitoring of mice (weight, vital signs, behavior and abdominal palpation) were carried out once a week. When the palpable tumor reached 100 mm^3^ (measured with calipers), 6 mice/group received a dose of vincristine (0.4 mg/kg) [26] or HCQ (60 mg/kg) or an association of vincristine and HCQ at the same concentration was injected daily for 9 consecutive days. Six control mice received (100 μL) of saline solution per day. Mice were euthanized at the onset of clinical signs or when bearing tumors of 1000 mm^3^ or at the end of the treatment.

Secondly, five millions of SK-N-DZ cells (100 μL) were injected s.c. in the left flank. Mice were treated with vincristine (0.4 mg/kg) or cisplatin (Mayne Pharma, Canada) i.p. at 8 mg/kg for 4 days [27, 28]. Mice receiving PBS at the same time were used as a negative control. In all in vivo experiments, mice were euthanized 10 days after the end of treatment or when tumor size reached 1000 mm^3^. Tumors were collected and immunohistochemistry with LC3B antibody was performed.

### Statistical analysis

Association tests were performed with the use of Fisher’s exact test. Spearman correlation values (rho) were used to compare the expression between LC3-II and pmTOR, pAKT and between Beclin 1 and pAKT. Statistical analyses were performed using GraphPad Prism 6 software. *P* values of less than 0.05 were considered to indicate statistical significance.

## Results

### Characteristics of patients

Clinicopathological characteristics of the 184 patients and tumors are detailed in (Table 1). These patients had a median follow-up period of 67 months (range newborn-174 months) and a median age at the time of diagnosis to be 26 months (range newborn-151 months).

**Table 1:** Patients clinical data

Variable		LC3B				Beclin-1			
	No (%)	No	%	Mean Intensity	*p*	No	%	Mean Intensity	*p*
All patients	184	148	80	0.83		153	83	1.03	
Age									
Median (Range), months	26 (0–151)								
<365 days	88 (48)	73	83	0.92		70	80	0.83	
≥365 days	96 (52)	74	77	0.75	ns	84	87.5	1.25	<0.01
Stage									
1	53 (29)	47	88	0.6	ns	48	90	1.30	ns
2	35 (19)	25	71	0.64		27	77	0.8	
3	23 (12)	21	91	0.94		20	86	1.14	
4	58 (32)	52	89	1.12		47	81	0.97	
4S	15 (8)	11	73	0.34	0.02	11	73	0.72	ns
MYCN status									
Amplified	14 (10)	13	87	0.81		11	73	0.87	
Non Amplified	127 (90)	103	81	0.50	ns	105	82	0.43	ns
Unknown	43								
Type of NB									
Standard	149 (81)	133	89	0.96		126	84	1.19	
Mass screening	35 (19)	29	82	0.8	ns	27	77	0.46	<0.001
Type of samples									
Primary tumors	184	148	80	0.83		153	83	1.03	
Metastases	47	41	87	0.78	ns	37	79	0.69	<0.01

### Autophagy and neuroblastoma

**Fig. 1:**
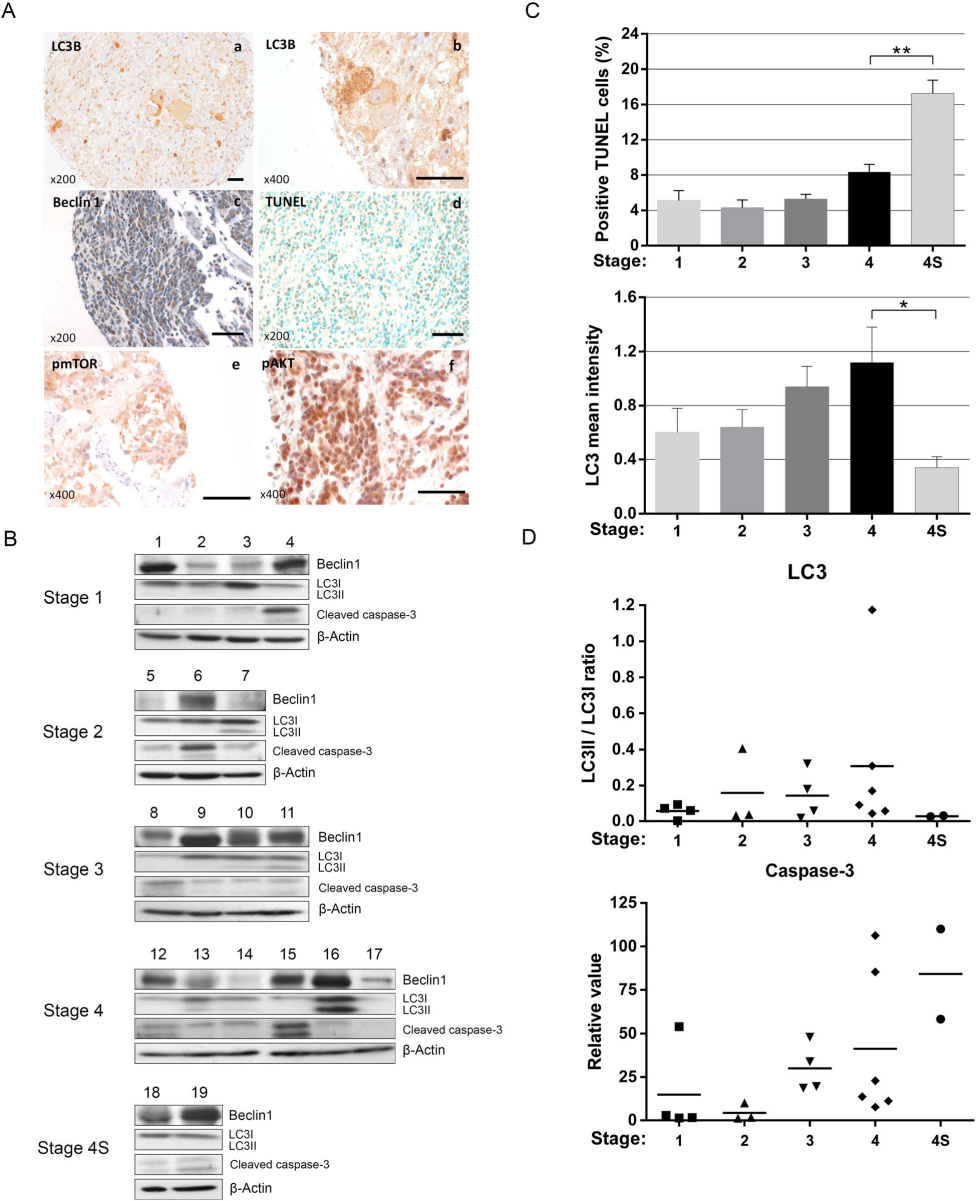
Evaluation of the level and the regulation of autophagy in NB and its correlation with apoptosis. **A** Autophagy was evaluated in TMA sections from NB tumor samples by immunohistochemistry using antibodies anti-LC3B (A.a,b) or anti-Beclin 1 (A.c). The expression levels of pmTOR (A.e) and pAKT (A.f), two autophagy-regulating proteins, were also studied on the same samples. Apoptosis was tested by TUNEL (A.d). Scale bars: 100 μm. **B** Immunoblotting analysis was performed on protein lysates from different frozen matched tumor samples with antibodies against either LC3B or Beclin 1 for autophagy and against cleaved caspase-3 for apoptosis. **C** The expression of LC3II and TUNEL positivity were semi-quantified according to the stage of tumors. **D** The ratio LC3II/LC3I and cleaved caspase-3 expression were evaluated by densitometry using tumor samples

The analysis of LC3 immunostaining clearly showed two types of positivity: uniform cytoplasmic staining, corresponding to LC3I (Fig. 1Aa) and a punctate cytoplasmic LC3-staining pattern specific to LC3II and therefore to autophagy (Fig. 1Ab). Furthermore, autophagy was frequent in NB (80% of the tumors) but with a very low intensity (mean intensity 0.83). These data were confirmed by Western blot analysis where LC3II expression was found in 12 out of 19 tumors (63%) (Fig. 1B). The cytoplasmic expression of Beclin 1 was also expressed in a majority of NB (83%) but with a low intensity (mean intensity 1.03) (Fig. 1Ac, Table 1). These data were confirmed by Western blot analysis showing Beclin 1 expression in 17 out of 19 tumors (90%) (Fig. 1B).

The LC3II expression was significantly higher in Stage 4 (mean intensity 1.12) than in Stage 4S (mean intensity 0.34) (Table 1, *p* = 0.02), confirmed by Western blot, where LC3II was undetectable in specimen from Stage 4S patients. Interestingly, the level of apoptosis detected by TUNEL (Fig. 1Ad) and by cleavage of caspase-3 was inversely correlated with autophagy, particularly in Stages 4 and 4S (Fig. 1C and D). However, expression levels of Beclin 1 were significantly higher in patients older than one year at diagnosis compared to younger patients (*p* < 0.001) (Table 1), in primary tumors than in metastasis (*p* < 0.01) and in standard NBs than in tumors coming from mass screening (*p* < 0.001), demonstrating that Beclin 1 is highly expressed in NB with poor prognosis. Among the major proteins present in the AKT pathway, there was a significant negative correlation between LC3II and pmTOR (Fig. 1Ae) (rho = −0.22, *p* < 0.01) as well as pAKT (rho = −0.22, *p* < 0.01) and between Beclin 1 and pAKT (Fig. 1Af) (rho = −0.13, *p* < 0.01), suggesting that autophagy is activated through AKT pathway inhibition.

### Chemotherapy induces autophagy in neuroblastoma cells

**Fig. 2:**
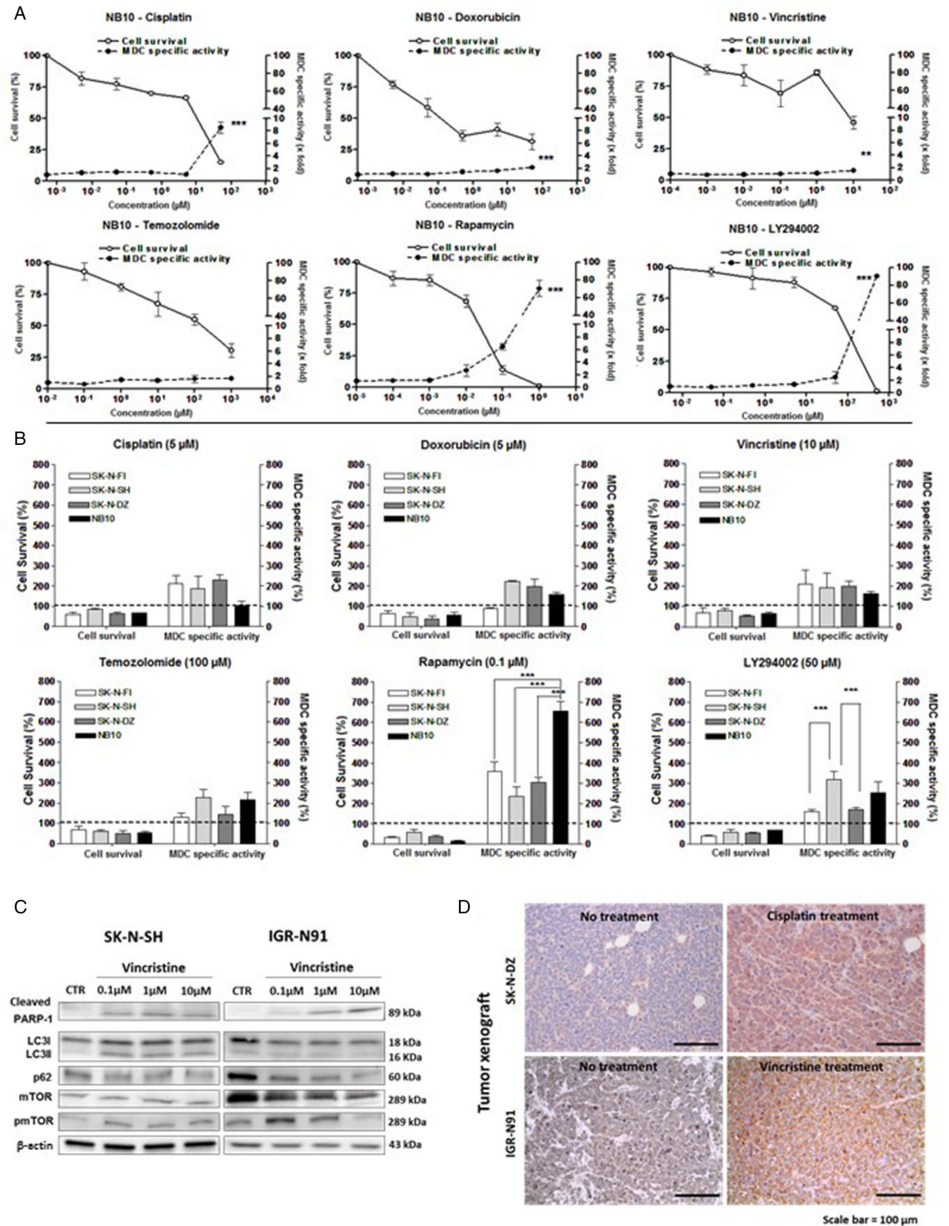
Chemotherapy induces autophagy in vitro and in vivo. The effect of chemotherapeutic agents on the autophagy of NB cell lines and tissues was analyzed. **a** NB-10 cells were treated with increasing concentrations of temozolomide, rapamycin, cisplatin, vincristine, doxorubicin or LY294002 for 24 h followed by MTT and MDC assays to measure cell viability and autophagy levels, respectively. Results are expressed as percentage of the corresponding control and represent mean ± SEM of 3 independent experiments (***: *P* < 0.001). **b** Cell viability and MDC specific autophagy were measured after IC50 treatment of different NB cell lines with chemotherapeutic agents. Results are expressed as percentage of the corresponding control and represent mean ± SEM of 3 independent experiments (***: *P* < 0.001). **c** Immunobloting analyses were performed on protein lysates from SK-N-SH and IGR-N91 cells treated with 0.1 to 10 μM of vincristine. Anti PARP-1, anti-LC3B, anti-p62, anti-mTOR, anti-pmTOR antibodies were used. β-actin was used as a loading control. **d** Tumor xenografts developed from SK-N-DZ or IGR-N91 cells were treated with cisplatin or vincristine. Slides were immunostained with anti-LC3B antibody. Scale bars: 100 μm

The formation of autophagosomes revealed by MDC indicates a significant increase of autophagy following treatment with increasing concentrations of cisplatin, vincristine and doxorubicin (Fig. 2a). Temozolomide didn’t induce high levels of autophagy even at high concentrations. AKT pathway inhibitors, such as LY294002 and rapamycin, were the most efficient, resulting in high autophagy levels (Fig. 2a). Following an IC50 concentration treatment, autophagy increased with all drugs in the four cell lines, independently to *MYCN* amplification (Fig. 2b). SK-N-SH and IGR-N91 cells incubated with vincristine for 24 h showed a moderate conversion of LC3I to LC3II suggesting that cells undergo apoptosis. In addition, p62 expression decreased when cells were treated with this drug demonstrating the presence of autophagy. This result was also confirmed by confocal analysis where assessment of LC3 showed a constant increase in cytoplasmic autophagosome formation in cells treated with vincristine (refer to Fig. 4c). This increased of autophagy was correlated with an increase of activated mTOR expression but only for the low concentration of vincristine (Fig. 2c). The induction of autophagy by chemotherapy was also found in in vivo experiments with strong LC3II positivity in tumors of NSG mice injected with SK-N-DZ and treated with cisplatin or IGR-N91 treated with vincristine (Fig. 2d).

### Inhibition of autophagy sensitizes NB cells to chemotherapy

**Fig. 3:**
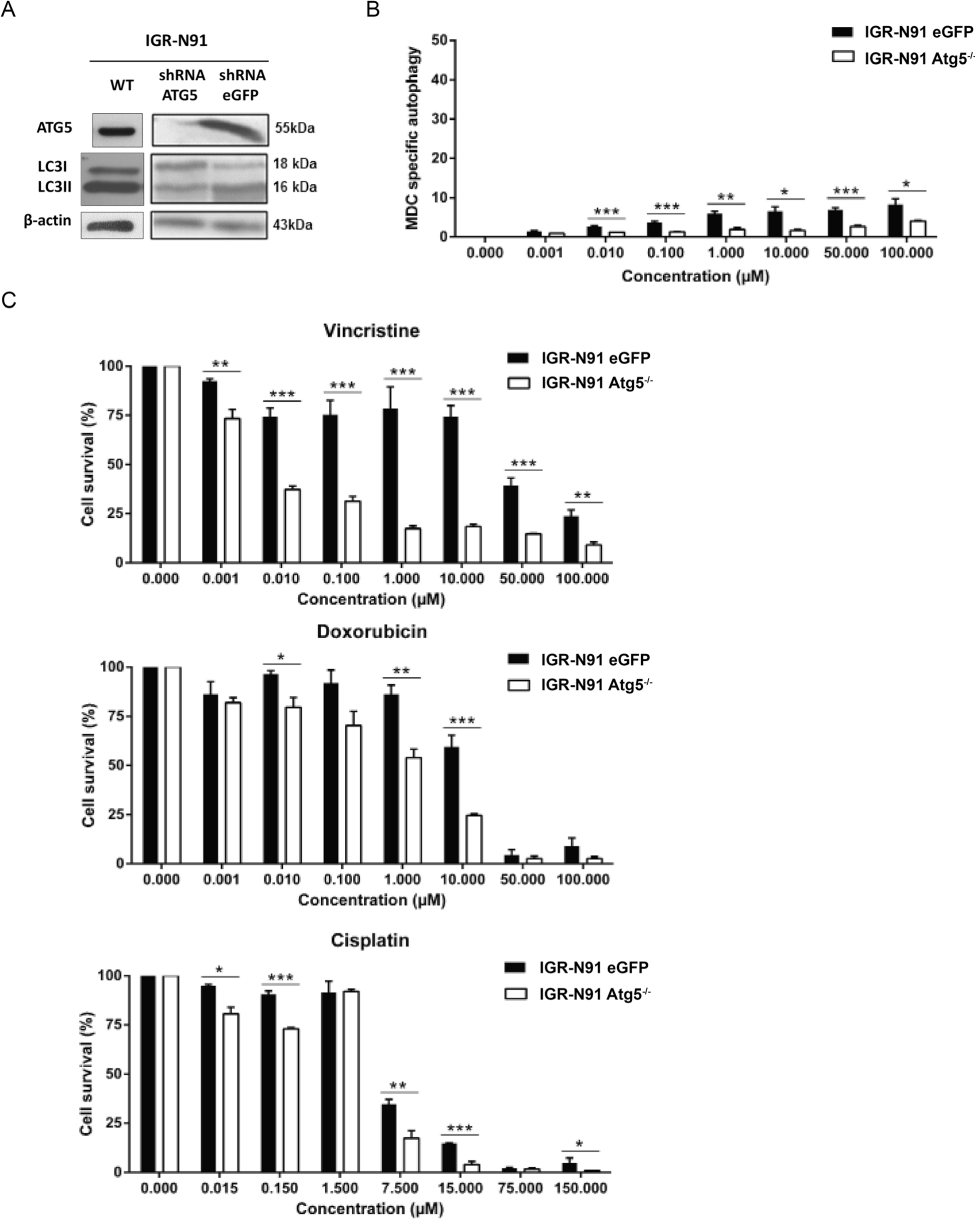
Inhibition of autophagy sensitizes NB cells to chemotherapy. IGR-N91 cells were transduced with ATG5-shRNA lentivirus to knockdown ATG5 gene expression and thereby inhibit autophagy or with eGFP-shRNA, used as a control. **a** Protein immunoblotting using anti-ATG5 and anti-LC3B antibodies was performed to demonstrate ATG5 gene knockdown and LC3 expression on IGR-N91-transduced cells or not (WT). β-actin was used as a loading control. **b** A MDC test for autophagy was performed on *ATG5* ^kd^ and control cells after treatment with increasing concentrations of vincristine. **c** Cell viability of *ATG5* ^kd^ and control cells was measured with a MTT assay following treatment with increasing concentrations of vincristine, doxorubicin or cisplatin. Results are expressed as percentage of corresponding control and represent mean ± SEM of 4 independent experiments (*: *P* < 0.05, ***: *P* < 0.001)

Transfection efficiency with lentivirus-based short hairpin RNA for *ATG5* knockdown (*ATG5* ^kd^) was confirmed by Western blot and by MDC (Fig. 3a and b). *ATG5* ^kd^ cells were significantly more sensitive to vincristine, doxorubicin or cisplatin than sheGFP cells (Fig. 3c), suggesting that autophagy contributes to chemoresistance in NB.

**Fig. 4:**
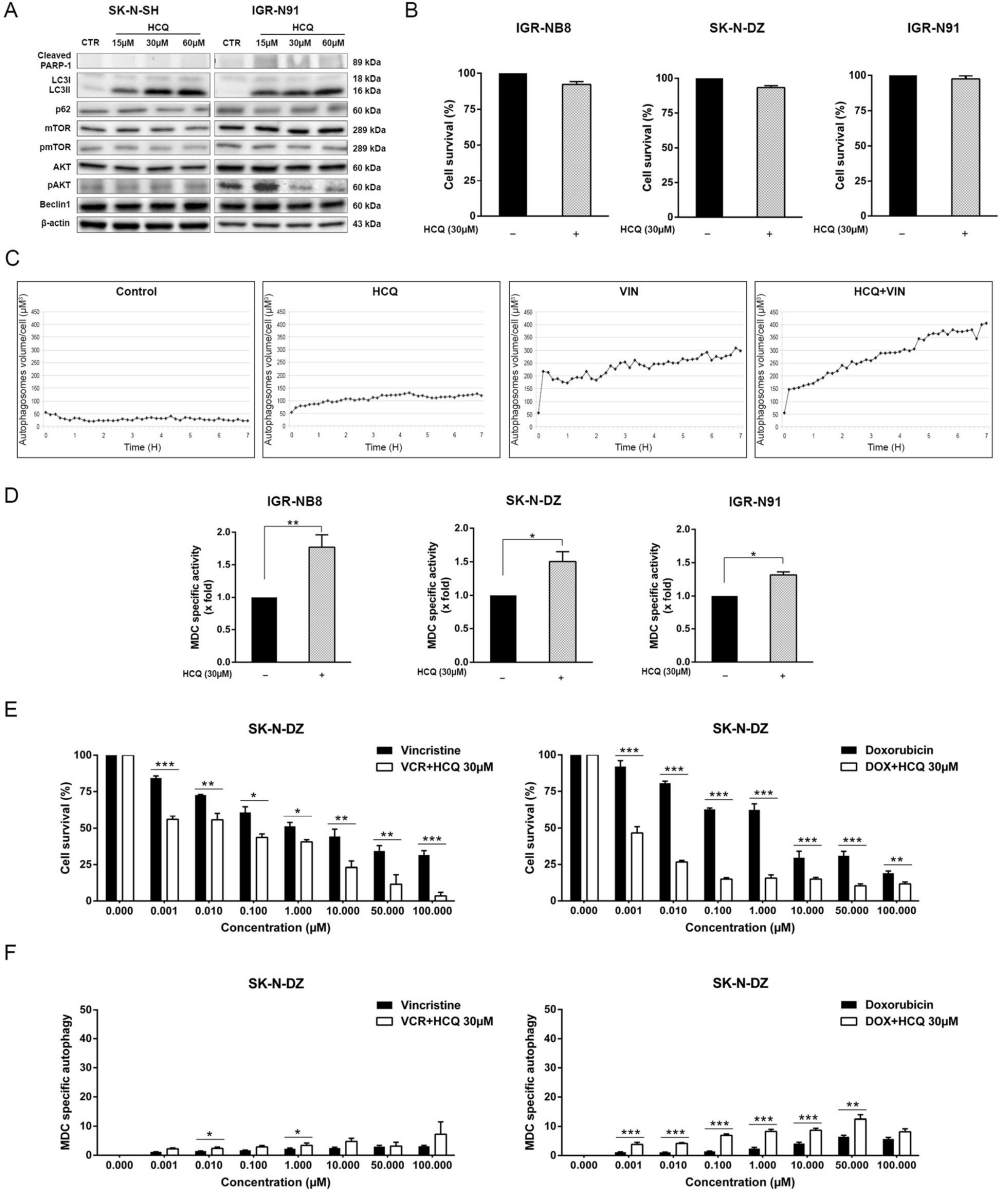
HCQ sensitizes NB cells to chemotherapy by inhibition of autophagy. **a** IGR-N91 and SK-N-SH cells were treated with 15 to 60 μM of HCQ and analyzed for LC3B, p62, mTOR, p-mTOR, AKT, pAKT, Beclin 1 protein expression by immunoblotting. β-actin was used as a loading control. **b** Cell viability of SK-N-DZ, IGR-N91 and IGR-NB8 was measured using an MTT assay pre and post HCQ treatment (30 μM). **c** Confocal microscopy analysis was done on transfected IGR-N91 cells with GFP-LC3 not treated or treated 7 h with HCQ 30 μM, vincristine 1 μM or the combination of the two drugs. **d** Autophagy in SK-N-DZ, IGR-N91 and IGR-NB8 cells was measured by an MDC assay pre and post HCQ treatment (30 μM). **e** SK-N-DZ cell viability was measured after vincristine or doxorubicin treatment combined or not with HCQ (30 μM). **f** The autophagic activity of SK-N-DZ cells was measured using a MDC agent after treatment with vincristine or doxorubicin, combined or not with HCQ (30 μM). Results are expressed as percentage of corresponding control and represent mean ± SEM of 4 independent experiments (*: *P* < 0.05, **: *P* < 0.01, ***: *P* < 0.001)

Treatment with HCQ induced an inhibition of the late stage of autophagy which was shown by an increase of the LC3II/LC3I ratio but no decrease of p62 in Western blot (Fig. 4a), as well as an accumulation of autophagosome detected by confocal microscopy (Fig. 4c and Additional file 2: Figure S2) and an increase of MDC positivity (Fig. 4d). The inhibition of autophagy was not correlated with increase of cell death (Fig. 4a and b). HCQ treatment does not modify the expression of pmTOR but decreases phosphorylated AKT levels at high concentrations (Fig. 4a).

The MTT test showed that cells treated with a combination of HCQ 30 μM and vincristine (0.001–100 μM) or doxorubicin (0.001–100 μM) were significantly more sensitive to chemotherapy than cells treated with vincristine or doxorubicin alone (Fig. 4e and Additional file 3: Figure S3A), confirming that HCQ increases sensitivity to chemotherapy. The MDC test also showed a dose-dependent increase of autophagosome numbers after treatment, enhanced by addition of HCQ, consistent with a late inhibition of autophagy (Fig. 4f and Additional file 3: Figure S3B).

### Autophagy inhibition decreases tumor progression in vivo

**Fig. 5:**
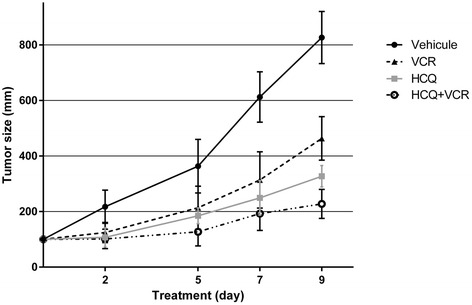
Effect of autophagy inhibition by HCQ on tumors in vivo*.* Six NSG mice of each group received a s.c injection of 5x10^6^ IGR-N91 cells. When tumors reached 100 mm^3^, mice were administered vincristine (0.4 mg/kg/day), HCQ (60 mg/kg), an association of vincristine and HCQ or vehicle (saline) for 9 days. The tumor size of the four groups of mice was compared during this period

To further investigate the effect of autophagy inhibition on tumor progression, we compared tumor development in mice treated or not with HCQ, vincristine or a combination of HCQ and vincristine. At the end of the treatment, the tumor volume was significantly lower in mice treated with HCQ and HCQ combined to vincristine than in control (*p* < 0.05 and *p* < 0.01 respectively). The association of HCQ and vincristine is significantly more efficient than vincristine alone (*p* < 0.05). Interestingly, HCQ used alone have a similar efficiency as vincristine alone (Fig. 5).

On another side, tumors were developed in 11/12 mice injected with sheGFP cells versus only 3/12 in mice injected with *ATG5* ^kd^ cells (Additional file 4: Figure S4).

## Discussion

Despite aggressive multimodal therapy, NB patients still have a poor prognosis, which is partially explained by chemoresistance of the tumor cells. Furthermore, it has been reported that autophagy is a potential mechanism that promotes tumor cell survival and confers chemoresistance [29]. In our study, we observed that autophagy is present at basal levels to maintain homeostasis. LC3 expression was not correlated to any clinicopathological data. However, autophagy was higher in Stage 4 tumors than 4S while apoptosis was lower in Stage 4 tumors than stage 4S, which could explain the aggressive properties of Stage 4 tumors compared to 4S. Beclin 1 is a factor of poor prognosis as it is highly expressed in tumors from NB patients older than one year old. In other cancers, such as human hypopharyngeal squamous cell carcinoma (HSCC), expression of Beclin 1 and LC3II correlates with poor prognosis [30]. Additionally, other studies showed that autophagy predicts resistance to chemotherapy and survival in melanoma [31]. We also observed a negative correlation between LC3II and pAKT as well as pmTOR and between Beclin 1 and pAKT, suggesting that autophagy activation occurs after inhibition of the AKT pathway since the inhibition of mTOR activates autophagy.

To determine whether autophagy is activated after chemotherapy treatment, three chemotherapeutic agents, used clinically for NB treatment, were studied: vincristine, doxorubicin and cisplatin. Our data demonstrate that the activation of autophagy by cisplatin and vincristine is dose-dependent. This is also observed with doxorubicin except in one cell line (SK-N-FI). These data were confirmed by in vivo study that show the activation of autophagy in NB tumors from mice receiving the cisplatin or vincristine treatment compared to non-treated tumors. In other cancers, doxorubicin is known as an inducer of the autophagosome formation in papillary thyroid cancer [32], whereas cisplatin induced autophagy in esophageal squamous cell carcinoma cells [33]. The *MYCN* gene amplification in several cell lines used in our study could change the state of autophagy indeed several studies have showed that overexpression of *CMYC* strongly induces autophagy in rat 3Y1 fibroblasts [33, 34]. Our data indicate that NB cell lines with amplified *MYCN* (SK-N-DZ, IGR-N91) didn’t show increased autophagy comparing to other cell lines with non-amplified *MYCN*. Temozolomide has been described as an inducer of autophagy [35]. In the present study, temozolomide doesn’t induce a dose-dependent increase of autophagy, detectable by MDC and by Western blot. Differently, the present study showed that LY294002 was a dose dependent activator of autophagy. This drug increases autophagy by inhibiting PI3K I, an element of the PI3K/AKT/mTOR inhibitory pathway to autophagy [36]. Rapamycin induces autophagy by inhibiting mTOR [37]. In the present study, an increase in autophagy detected by MDC was associated with treatment by rapamycin in the four studied NB cell lines. It has been described that in the presence of rapamycin, the dephosphorylation of many proteins activates the transcription of ATG8 and ATG14 genes, known to be associated with autophagy [13].

Beclin 1 has many cell functions, such as inducing autophagosome formation and acting as a tumor suppressor [38]. It is a mediator of various cellular cascades, including autophagy, apoptosis and cell differentiation [39]. The association of certain genes of the Bcl-2 family with Beclin 1 inhibits the autophagic function by limiting its partnering with a class III PI3K and activates its apoptotic function instead [40]. It is the positive regulation of Beclin 1 that induces the autophagic and differentiation cascades [39]. Also, Beclin 1 loses its autophagic abilities and induces apoptosis when there is an increased activity of caspase-9 [41]. This caspase has the property of cleaving Beclin 1 and creating a C-terminal fragment promoting pro-apoptotic activity [41]. According to some studies, doxorubicin activates caspase-9 and positively regulates the expression of Beclin 1 [41]. Consequently, this particular NB drug induces apoptosis.

Collectively, our data demonstrate that autophagy is activated in response to chemotherapy in NB cells but has not answered if this autophagy is an autophagy-mediated cell death or autophagy-mediated cell survival (chemoresistance). We investigated the effect of combining chemotherapy with autophagy inhibition. In the early stages of carcinogenesis, autophagy acts as a primary tumor suppressor and inhibits tumor progression [21]. However, once tumor is established, autophagy tend to be a protective mechanism used by tumors cells to overcome chemotherapy and other metabolic stress. We used HCQ to target the late stages of autophagy by blocking the fusion of autophagosomes with lysosomes [42]. Interestingly, mice treated with HCQ alone have decreased tumors volume compared to mice treated with vincristine. This results support some clinical trials that use HCQ alone to treat patients with pancreatic cancer, breast cancer, renal cell carcinoma or chronic lymphocytic leukemia [23, 43]. In the present study, HCQ was used at non-toxic concentrations that trigger autophagy inhibition. Our data demonstrated that mice treated with a combination of vincristine and HCQ develop tumors with a significantly less extend than mice treated with vincristine alone. These data demonstrate, for the first time, that inhibition of autophagy using HCQ sensitizes NB cells to classical chemotherapy supporting the idea that autophagy acts as a cytoprotective mechanism [44] and its inhibition may promote apoptosis in cancer cells [45]. Several studies and clinical trials have investigated autophagy inhibition using different pharmacological agents, usually in combination with chemotherapy, radiotherapy or other targeted anti-cancer therapies [46]. Some data demonstrated a significant increase in long-term survival when the treatment includes CQ [47]. Reports from clinical trials indicate that when CQ was added to conventional therapy, improvement of mid-term survival for glioblastoma multiform patients was seen [48]. Taken together, these results confirm that autophagy promotes chemoresistance and its inhibition sensitizes NB cells to chemotherapy.

## Conclusions

Our study demonstrates that autophagy is present in NB tumors at a basal level and it is activated after chemotherapeutic that confers chemoresistance. The use of HCQ sensitizes cells to the conventional chemotherapy in NB treatment. Therefore, we propose that HCQ could be used as an adjuvant therapy in clinical trials to enhance the efficacy of chemotherapy in NB patients.

## Additional files

Additional file 1: Figure S1. Control for immunogistochemistry. Normal mouse or rabbit IgG at the same concentration as the primary antibody were used as negative control (NC) and synaptophysin (1/100, Polyclonal, SP11, Thermofisher Scientific) as positive control (PC). (PDF 379 kb)
Additional file 2: Figure S2. GFP-LC3 transfection and confocal microscopy. The cell line IGR-N91 transfected with GFP-LC3 were not treated (a), treated with vincristine alone (1 μM) (b), with HCQ (30 μM) alone (c) or with the association of the two drugs (d) and then analyzed with confocal microscopy for 7 h. Treatment with HCQ induced an inhibition of the late stage of autophagy which was shown an accumulation of autophagosome. (PDF 789 kb)
Additional file 3: Figure S3. HCQ sensitizes NB cells to chemotherapy by inhibition of autophagy. A. N91-IGR or NB8-IGR cell viability was measured after vincristine or doxorubicin treatment combined or not to HCQ 30 µM. Results are expressed as percentage of corresponding control and represent mean ± SEM of 4 independent experiments. B. Autophagic activity of N91-IGR or NB8-IGR was measured using MDC agent after increasing concentration of vincristine or doxorubicin treatment combined or not to HCQ 30 µM. Fluorescence was quantified by spectrophotometer. (*: *P* < 0.05), (**: *P* < 0.01), (***: *P* < 0.001). (PDF 215 kb)
Additional file 4: Figure S4. Effect of ATG5 knockdown xenografts on tumor growth. (11/12) NSG Mice developed xenograft tumor from s.c injection of 5.10^6^ sheGFP cells and only (3/12) from shATG5 cells (Table A). When tumor reached 200 mm3, mice were administered vincristine (0.4 mg/kg/day) or vehicle (saline) for 5 days. Progression of tumor volume was followed in each group. Each data point represents the mean 6 ± SE tumor volume for sheGFP cells and 1 or 2 for shAtg5 cells. At day 6, Tumors size from shATG5 cells were reduced compared with tumors from eGFP (B). Also, tumors from shATG5 cells were more sensitive to vincristine comparing to control cells. Immunohistochemistry was performed on the sections of tumors developed in the mouse model. with LC3 antibody and showed a high expression of LC3 II in control cells and a very low in cells ATG5 knockdown as well as treated (b, d) or not treated (a, c) (C). (PDF 259 kb)

## Abbreviations

ATG: Autophagy-related genes
Cis: Cisplatin
CQ: Chloroquine
DXR: Doxurubicin
HCQ: Hydroxycholoroquine
IHC: Immunohistochemistry
LC3: Microtubule-associated protein light chain 3
MDC: Monodansylcadaverine
MTT: 3-(4,5-dimethylthiazol-2-yl)-2,5-diphenyltetrazolium bromide
NB: Neuroblastoma
NSG: NOD scid gamma
shRNA: Short-hairpin RNA
TMA: Tissue microarray
VCR: Vincristine
