# Supplementary figures and images for: Autophagy is associated with chemoresistance in neuroblastoma
Source: BMC Cancer. 2016 Nov 15;16:891. doi: 10.1186/s12885-016-2906-9 (PMC5109645; doi:10.1186/s12885-016-2906-9)

**Figure S1**

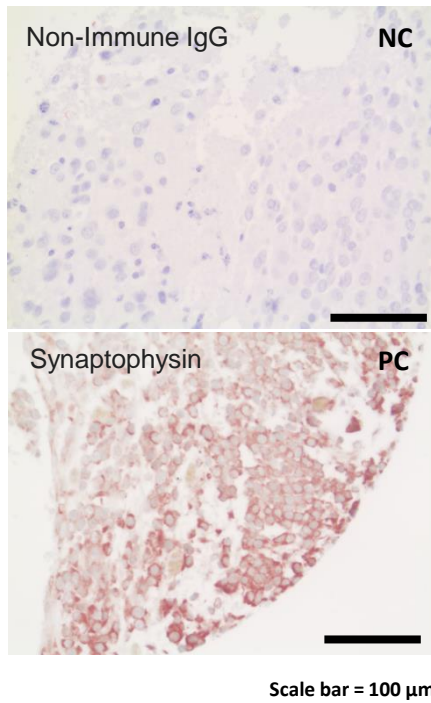

NC: Negative control

PC: Positive control

Supplement: Additional file 1: Figure S1. — Control for immunogistochemistry. Normal mouse or rabbit IgG at the same concentration as the primary antibody were used as negative control (NC) and synaptophysin (1/100, Polyclonal, SP11, Thermofisher Scientific) as positive control (PC). (PDF 379 kb) [file 12885_2016_2906_MOESM1_ESM.pdf]

A)

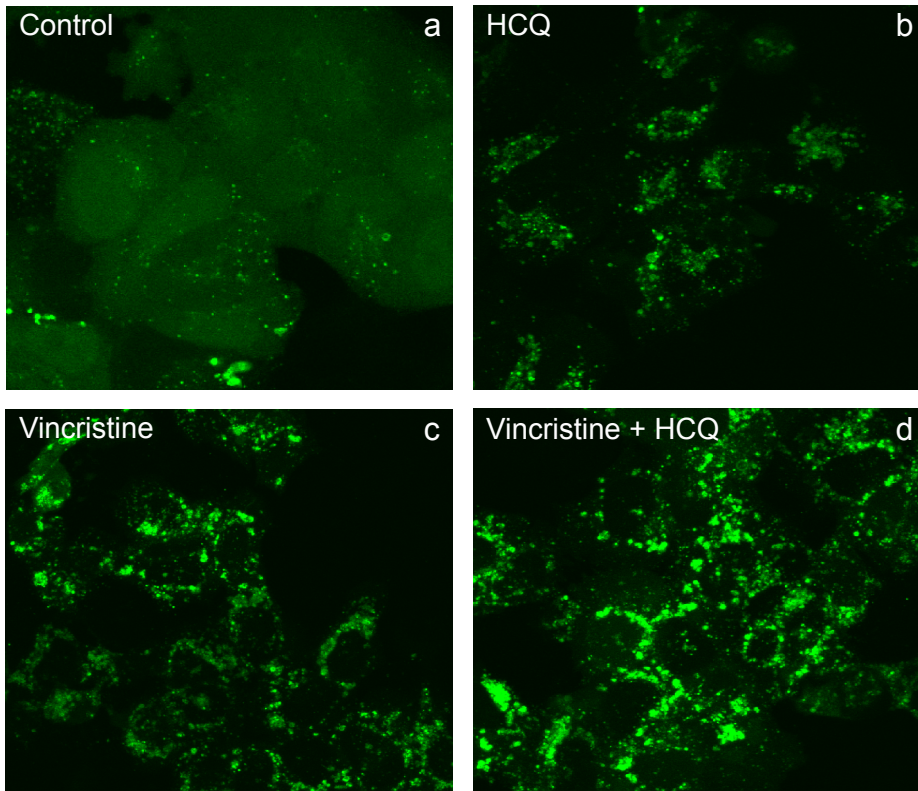

Supplement: Additional file 2: Figure S2. — GFP-LC3 transfection and confocal microscopy. The cell line IGR-N91 transfected with GFP-LC3 were not treated (a), treated with vincristine alone (1 μM) (b), with HCQ (30 μM) alone (c) or with the association of the two drugs (d) and then analyzed with confocal microscopy for 7 h. Treatment with HCQ induced an inhibition of the late stage of autophagy which was shown an accumulation of autophagosome. (PDF 789 kb) [file 12885_2016_2906_MOESM2_ESM.pdf]

A)

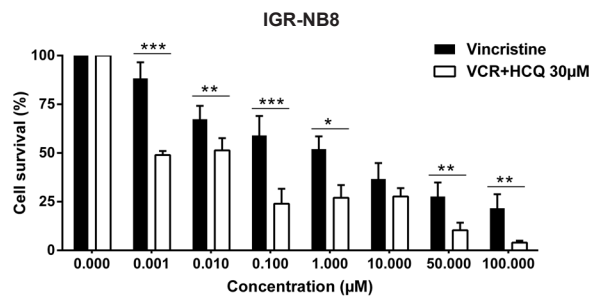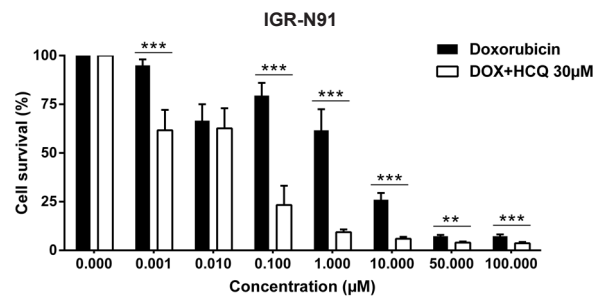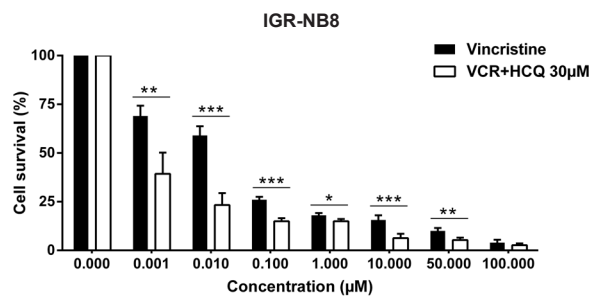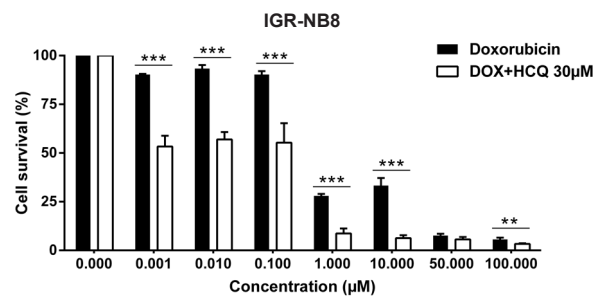

B)

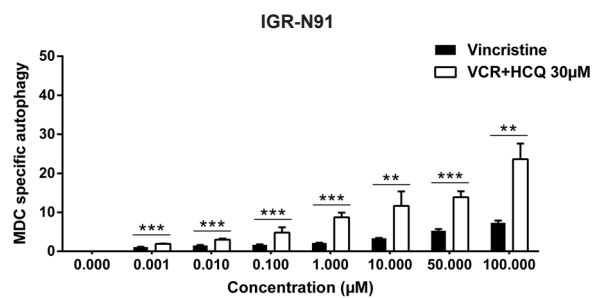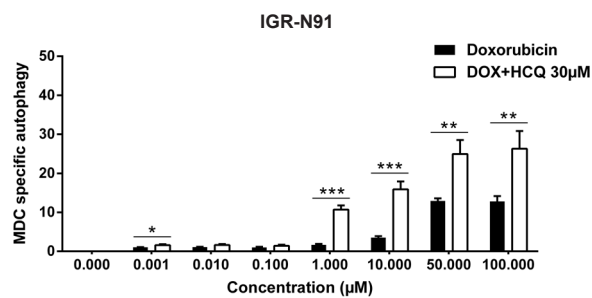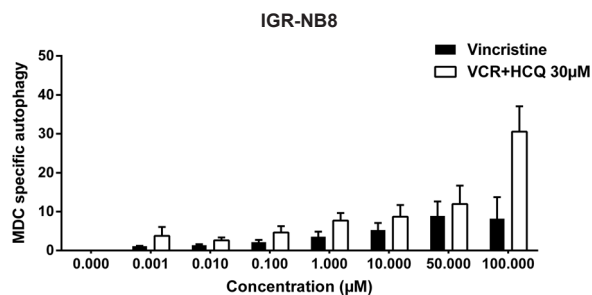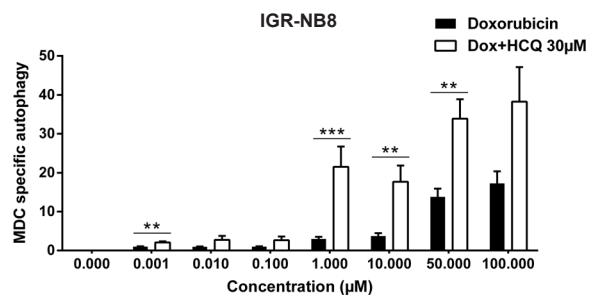

Supplement: Additional file 3: Figure S3. — HCQ sensitizes NB cells to chemotherapy by inhibition of autophagy. A. N91-IGR or NB8-IGR cell viability was measured after vincristine or doxorubicin treatment combined or not to HCQ 30 µM. Results are expressed as percentage of corresponding control and represent mean ± SEM of 4 independent experiments. B. Autophagic activity of N91-IGR or NB8-IGR was measured using MDC agent after increasing concentration of vincristine or doxorubicin treatment combined or not to HCQ 30 µM. Fluorescence was quantified by spectrophotometer. (*: P < 0.05), (**: P < 0.01), (***: P < 0.001). (PDF 215 kb) [file 12885_2016_2906_MOESM3_ESM.pdf]
