# Supplementary material for: Autophagy is associated with chemoresistance in neuroblastoma
Source: BMC Cancer. 2016 Nov 15;16:891. doi: 10.1186/s12885-016-2906-9 (PMC5109645; doi:10.1186/s12885-016-2906-9)
Supplement: Additional file 4: Figure S4. — Effect of ATG5 knockdown xenografts on tumor growth. (11/12) NSG Mice developed xenograft tumor from s.c injection of 5.106 sheGFP cells and only (3/12) from shATG5 cells (Table A). When tumor reached 200 mm3, mice were administered vincristine (0.4 mg/kg/day) or vehicle (saline) for 5 days. Progression of tumor volume was followed in each group. Each data point represents the mean 6 ± SE tumor volume for sheGFP cells and 1 or 2 for shAtg5 cells. At day 6, Tumors size from shATG5 cells were reduced compared with tumors from eGFP (B). Also, tumors from shATG5 cells were more sensitive to vincristine comparing to control cells. Immunohistochemistry was performed on the sections of tumors developed in the mouse model. with LC3 antibody and showed a high expression of LC3 II in control cells and a very low in cells ATG5 knockdown as well as treated (b, d) or not treated (a, c) (C). (PDF 259 kb) [file 12885_2016_2906_MOESM4_ESM.pdf]

A)

Cells injected	Developed tumors	Tumor latency (days)	Treatment 5 days	Number of tumors/group	Tumor volume (mm3) at day 6
**SheGFP Cells**	11/12	34.5	Saline	6	1392.1
			Vincristine (0.4 mg/kg/day)	5	891.6
**ShATG5 (*ATG5*^{kd}) Cells**	3/12	36.3	Saline	2	883.9
			Vincristine (0.4 mg/kg/day)	1	659.3

B)

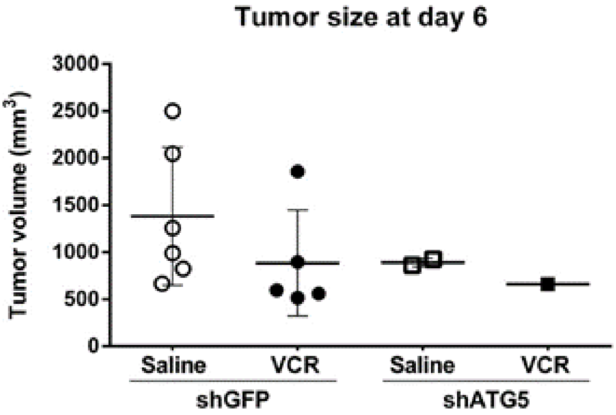

C)

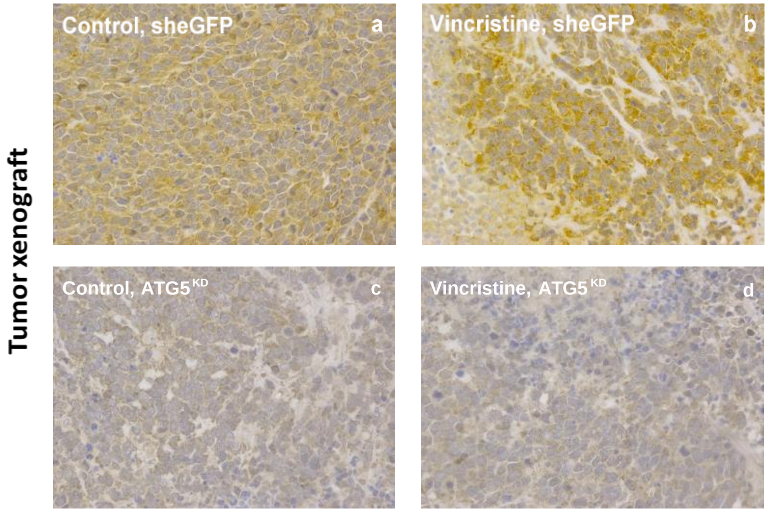

anti-LC3B staining
